# GDM-Induced Macrosomia Is Reversed by Cav-1 via AMPK-Mediated Fatty Acid Transport and GLUT1-Mediated Glucose Transport in Placenta

**DOI:** 10.1371/journal.pone.0170490

**Published:** 2017-01-26

**Authors:** Guo Yao, Yafang Zhang, Di Wang, Ruirui Yang, Hui Sang, Linlin Han, Yuexia Zhu, Yanyan Lu, Yeke Tan, Zhanping Shang

**Affiliations:** 1 Department of Pediatrics, Qilu Hospital of Shandong University, Jinan, China; 2 Department of Pediatrics, Taian City Central Hospital, Taian, China; 3 Department of Pathophysiology, Taishan Medical University, Taian, China; 4 Taian City Central Hospital, Taian, China; CHA University, REPUBLIC OF KOREA

## Abstract

**Objective:**

To investigate if the role of Cav-1 in GDM-induced macrosomia is through regulating AMPK signaling pathway in placenta.

**Methods:**

We used diagnostic criteria of gestational diabetes mellitus (GDM) and macrosomia to separate and compare placental protein and mRNA levels from GDM with macrosomia group (GDMM), GDM with normal birth weight group (GDMN) and normal glucose tolerance (NGT) with normal birth weight group (CON). Western blotting was performed to examine differentially expressed proteins of caveolin-1 (Cav-1) and Adenosine monophosphate-activated protein kinase (AMPK) signaling pathway related proteins, including phosphorylated-AMPKα(Thr172), AMPKα, phosphorylated-Acetyl-CoA carboxylase(Ser79) (p-ACC(Ser79)), ACC and glucose transporter 1 (GLUT1) in placenta between the three groups. The mRNA levels of Cav-1, AMPKα, ACC and GLUT1 in placenta were measured by real time-PCR.

**Results:**

In the GDMM placenta group, both protein and mRNA levels of Cav-1 were down-regulated, while GLUT1 was up-regulated; the phosphorylation and mRNA levels of ACC and AMPKα were decreased, but total ACC protein levels were increased compared to both the GDMN (p<0.05) and CON groups (p<0.05). In GDMM placenta group, there was a significant negative correlation observed between neonatal birth weight (NBW) and protein expression levels of Cav-1, p-ACC(Ser79) and p-AMPKα(Thr172) (p<0.05), while positive relationship with ACC and GLUT1 protein levels. Besides, in GDMM group placental mRNA levels, NBW had a positive correlation with GLUT1 (p<0.05), while negative with Cav-1, AMPKα and ACC expression (p<0.05). Cav-1 protein expression was positively associated with p-AMPK and p-ACC (p<0.05), and negatively associated with GLUT1 (p<0.05). Interestingly, p-AMPK protein expression was closely related to p-ACC (p<0.05), but not with GLUT1.

**Conclusion:**

GDM-induced macrosomias have more severe inhibition of Cav-1 expression in placenta. Cav-1 is associated with placental glucose and fatty acid transport via the induction of AMPK signaling pathway and the reduction of GLUT1 signaling pathway to reverse GDM-induced macrosomia.

## Introduction

GDM, which is defined as a group of glucose and lipid metabolism disorders [1], severely threats fetal perinatal period and growth of offspring in the long term [2–4]. It is generally considered that the neonates born to diabetic mothers have an increased risk for macrosomia. Macrosomia is accompanied by more risks of dystocia, neonatal asphyxia, neonatal hypoglycemia and perinatal death. Besides, many studies confirme that GDM-induced macrosomia has the long term risk of diabetes, obesity and other metabolic dysfunctions later in life [5, 6].

Previous studies confirme that macrosomia fails to effectively reduce despite diabetic mothers have acceptable glycemic index control [7]. Alterations of placental protein induced by GDM are believed to play a critical role in the transport of glucose and fatty acid to fetal [8]. Collectively, it is conceivable that fetal growth is largely determined by expression of proteins related to placental glucose and fatty acid metabolism, in addition to maternal blood glucose levels.

AMPK signaling pathway is a new research direction of glucose and fatty acid metabolism [9]. The significantly decreased protein expression level of p-AMPK in GDM placenta is associated with macrosomia [10], but the underlying mechanism is unclear [11]. Increasing evidence suggests that Cav-1, as the hub of the signaling pathway, plays an important role in the development of diabetes through regulating glucose and fatty acid metabolism [1, 12–15]. Cav-1 widely involves in AMPK signaling pathway via increasing phosphorylation of ACC to inhibit the cellular fatty acid synthesis [16]. In skeletal muscle cells, phosphorylation of AMPK can be promoted by exercise via Cav-1, resulting in inducement of GLUT4-mediated glucose uptake [17]. Similar regulation also exists in diabetic myocardial cells [18], however, there is a lack of relevant evidence regarding the regulatory role of Cav-1 in participating in GLUT1-mediated glucose metabolism and ACC-mediated fatty acid metabolism via AMPK signaling pathway in placenta of GDM-induced macrosomia.

Concerning the crucial role of Cav-1 in glucose and fatty acid metabolism, and the clinical significance of GDM induced-macrosomia, our study highlights the importance of Cav-1 in placental glucose and fatty acid transports via AMPK signaling pathway, to investigate the role of Cav-1 in GDM induced-macrosomia and provide a new perspective on prevention and treatment of GDM induced-macrosomia.

## Materials and Methods

### Ethics statement

This study was approved by the research ethical committee of Taishan Medical University and written informed consent was provided by all volunteers.

### Subjects

According to the unified GDM diagnosis criteria of the international diabetes and pregnancy study group (IADPSG) [19], the pregnancy woman were divided into GDM group (n = 26) and NGT group (n = 26), age 23–40 years old. Placentas of the neonate were classified into three groups based on the diagnostic criteria of macrosomia, consisting of GDMM group (n = 13), GDMN group (n = 13) and randomly selected CON group (n = 13).

### Sample collection

Placentas were received from pregnant women vaginal delivery or cesarean sections. To reduce the degradation of RNA and protein, approximately 1cm^3^ of placental tissue were obtained from the fetal surface immediately following delivery. After removed, the tissue was quickly washed with ice-cold NS. Then placed in marked frozen tube to store at -80°C for further protein and mRNA extraction.

### Western blotting

Western blotting on Cav-1, GLUT1, p-ACC(Ser79), ACC, AMPKα and p-AMPKα(Thr172) was performed on placental tissues from GDMM, GDMN, CON groups. Homogenized samples were extracted with the mixture consisted of 99% of radioimmunoprecipitation assay (RIPA) and 1% of phenylmethylsulfonyl fluoride (PMSF), Bicinchonininc acid (BCA) was used to protein quantification. Total protein were denatured by boiling at 100°C for 5 min following adding protein 4 × Buffer. 12% resolving and 4% stacking polyacrylamide gels (Solarbio Bio Inc., Shanghai, CHINA) with a Bio-Rad system (Bio-Rad, Shanghai, USA) were used to isolate target protein from 40ug of protein samples. Then, protein proteins were transferred to PVDF membranes at 80V for 60 min in ice cold transferring buffer and blocked in 10% skimmed milk at 4°Covernight. The PVDF membranes were incubated with different antibodies at 4°Covernight: Rabbit polyclonal to Glucose Transporter GLUT1 (Abcam, ab14683, USA, 1:1800), rabbit Anti-Cav-1(SANTA CRUZ Bio Inc, sc-894, USA, 1:1500), ACC rabbit mAb (Cell Signaling Technology Inc, #3676, USA, 1:800), anti-p-ACC(Ser79) rabbit mAb (Cell Signaling Technology Inc, 11818S, USA, 1:800), AMPKα rabbit mAb (Cell Signaling Technology Inc, 2603S, USA, 1:1200), p-AMPKα(Thr172) rabbit mAb (Cell Signaling Technology Inc, 2535, USA, 1:1200) and rabbit anti-GAPDH (Cwbio, CW0101M, CHINA, 1:1200). and incubated with Goat Anti-Rabbit IgG, AP Conjugated (Cwbio, CW0111S, CHINA, 1:2200) for 2.5 h at least at room temperature. Using the BCIP/NBT Alkaline Phosphatase Color Development Kit (Cwbio Bio Inc., Beijing, CHINA) indicated the positive target strips. The relative protein expression used VisionWorks LS of BioSpectrum Imaging System.

### RNA extraction and cDNA synthesis

Total RNA was extracted from 60 mg of placental tissue using the TRIzol method (Invitrogen, Life technologies, USA), using a NanoDrop spectrophotometer (Thermo Fisher Scientific, Waltham, MA) quantified RNA concentration. 20ul of total reaction system was reverse transcribed to cDNA with the SuperRT cDNA Synthesis Kit (Cwbio Bio Inc., Beijing, CHINA) in Eppen-dorf Thermocycler (Eppendorf AG, Hamburg, Germany), consisted of 4ul of dNTP Mix and 5×RT Buffer, 2ul of Primer Mix, 1ul of SuperRT and sample RNA and 8ul of RNase-Free Water briefly. The thermocycling parameters: 50 min at 42°C, 5 min at 85°C.

### Real-time polymerase chain reaction (RT-PCR)

Cav-1, GLUT1, ACC and AMPKα mRNA expression levels in placenta from GDMM, GDMN, CON groups were determined by real-time polymerase chain reaction. Primers sequences were as follow: 5’-TGGCATCAACGCTGTCTTCT-3’ (GLUT1-F), 5’-CTAGCGCGATGGTCATGAGT-3’ (GLUT1-R), 5’-ACCTCAACGATGACG TGGTCAAGA-3’ (Cav-1-F), 5’-TGGAATAGACACGGCTGATGCACT-3’ (Cav-1-R), 5’-GCT GCTCGGATCACTAGTGAA-3’ (ACC-F), 5’-TTCTGCTATCAGTCTGTCCAG-3’ (ACC-R), 5’-ACTGTACCAGGTCATCAGTACACC-3’ (AMPKα-F), 5’-CCACCATATGCCTGTGACAA -3’ (AMPKα-R), 5’-GAAGATGGTGATGGGATTTC-3’ (GAPDH-F), 5’-GAAGGTGAAGGTC GGAGTC-3’ (GAPDH-R). RT-PCR was performed on cDNA using LightCycler 96 Real-Time PCR System with UltraSYBR Mixture (With ROXI) (Cwbio Bio Inc., Beijing, CHINA). Each sample was configured to a 25ul reaction volume, and GAPDH was used as reference gene. Reaction conditions were as follows: pre-denaturation at 95°C for 10 min, followed by 45 cycles of denaturation at 95°C for 30 sec, annealing at 52°C for 40 sec and extension at 72°C for 40sec. To ensure primer specificity, we set a melt-curve after the 45 amplification cycles (95°C for 5 sec, 65°C for 60 sec and 97°C for 1 sec).

### Statistics

The clinical characteristics datas and differential expression of placental protein and mRNA were input and analyzed by GraphPad Prism 5.0 database. All results were presented as mean±SD, *P*<0.05 was considered as significant. The differences of baseline clinical datas between GDM and NGT group were assessed by one-sample t test. The placental mRNA and protein expression in three groups were analyzed using the single factor analysis of variance (ANOVA) analysis followed by Student-Newman-Keuls. Pearson correlation analysis was used to measure the relationship between NBW and the levels of mRNA and proteins, and the relationship of the protein exepression of Cav-1 to p-AMPKα(Thr172), p-ACC(Ser79), GLUT1.

## Results

### General characteristics of pregnant women

Maternal baseline characteristics are displayed in Table 1. In the second trimester, fasting blood glucose (FBG) levels in GDM group was higher 1.01mmol/l than NGT group (p<0.001). While, no obvious difference of FBG was observed in the third trimester (p>0.05). Moreover, there were no significant differences of maternal age, height, primipara and blood pressure between GDM and NGT group (p>0.05). In addition, two cases of family history of diabetes existed in GDM group, but not in NGT group.

**Table 1:** Maternal Baseline clinical characteristics of the research population.

	NGT	GDM	
	(n = 26)	(n = 26)	*P*
Age (year)	28.52±3.40	29.76±4.50	0.345
Height (cm)	160.90±4.67	162.40±4.36	0.273
Primipara	16 (61.5%)	15 (57.7%)	
Family history of diabetes (n, %)	0 (0%)	2 (7.7%)	
Blood pressure			
Systolic pressure (mmHg)	118.42±10.96	119.71±11.00	0.804
Diastolic pressure (mmHg)	77.96±7.63	76.12±8.58	0.196
Maternal fasting glucose			
The second trimester(mmol/l)	4.62±0.24	5.63±0.70	0.000
The third trimester (mmol/l)	4.46±0.24	5.09±1.15	0.121

### Neonatal characteristics

The neonatal examination results checked within one hours after birth was shown on Table 2. Compared with NGT group, NBW in GDM group was significant higher (p = 0.0002). 13 cases of macrosomia were all born in GDM group. Equally, the average neonatal birth length (NBL) in GDM group was also at a higher level (p = 0.009). While, the neonates of two groups had no statistic differences in gestational age, gender proportion, placental weight and umbilical cord length.

**Table 2:** Neonatal characteristics.

	NGT	GDM	
	(n = 26)	(n = 26)	*P*
Gestational Age (week)	39.78±1.50	39.46±1.19	0.417
Gender			
Male (n, %)	15, 58	14, 54	
Female (n, %)	11, 42	12, 46	
Neonatal birth Weight (g)	3120±168.5	3974±390.2	0.0002
Macrosomia (n, %)	0 (0)	13 (50)	
Birth Length (cm)	50.96±0.88	53.40±1.56	0.009
Placental weight (g)	509.1±47.86	525.2±62.66	0.326
Umbilical cord length (cm)	54.35±6.96	55.76±5.85	0.449

### Correlation between NBW and maternal FBG

**Fig 1:**
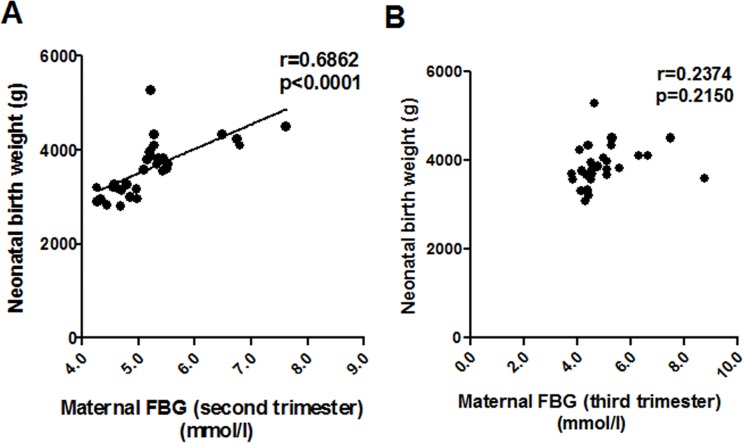
The relationship between maternal fasting blood glucose (FBG) levels and neonatal birth weight (NBW). (A) Pearson correlation analysis of the relationship between maternal FBG (second trimester) and NBW. (B) Correlation analysis between maternal FBG (third trimester) and NBW.

An positive correlation between NBW and maternal FBG (second trimester) was found in all patients (r = 0.686; p = 0.000; Fig 1A). While, we could not observe any correlation between levels of NBW and maternal FBG (third trimester) (r = 0.2374; p = 0.2150; Fig 1B).

### Alterations of protein levels of Cav-1 and AMPK signaling pathway related proteins

**Fig 2:**
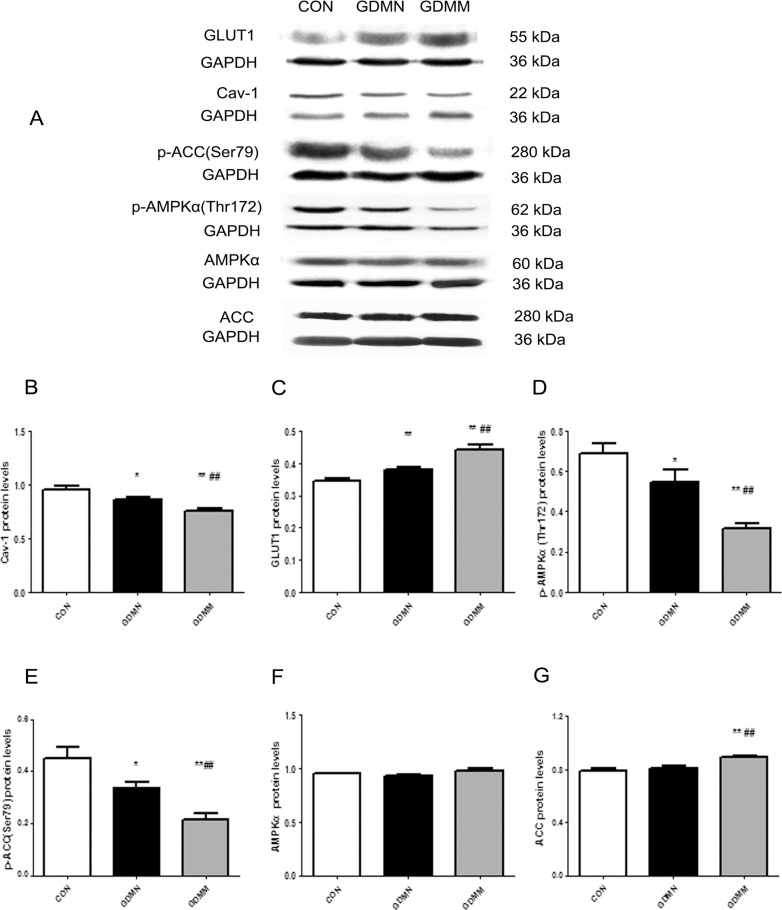
Protein levels of Cav-1, GLUT1, p-ACC(Ser79), p-AMPKα(Thr172), AMPKα and ACC in placenta. (A) A representative image of Western blots for GLUT1, Cav-1, p-ACC(Ser79), p-AMPKα(Thr172), AMPKα and ACC in placenta from each group. GAPDH was used to verify equivalent loading. (B-G) Graphic representation of relative abundance of Cav-1, GLUT1, p-AMPKα(Thr172), p-ACC(Ser79), AMPKα and ACC normalized to GAPDH. Data are presented as Mean ± SD of thirteen samples from each group. *p<0.05, **p<0.01 vs CON group; #p<0.05, ##p<0.01 vs GDMN group.

To explore the role of Cav-1 in the metabolism of glucose and fatty acid in placenta, we detected alterations of proteins expression of Cav-1 and AMPK signaling pathway related proteins in GDMM, GDMN and CON groups. Results were shown in Table 3 and Fig 2.

**Table 3:** Protein levels of Cav-1, GLUT1, p-AMPKα(Thr172), p-ACC(Ser79), AMPKα and ACC in placental tissue.

	Cav-1	GLUT1	p-AMPKα(Thr172)	p-ACC (Ser79)	AMPKα	ACC
	(n = 13)	(n = 13)	(n = 13)	(n = 13)	(n = 13)	(n = 13)
CON	0.96±0.13	0.34±0.03	0.69±0.18	0.45±0.04	0.94±0.03	0.79±0.07
GDMN	0.86±0.06*	0.38±0.02**	0.55±0.21*	0.33±0.02*	0.93±0.06	0.81±0.07
GDMM	0.76±0.07**##	0.44±0.06**##	0.32±0.08**##	0.22±0.03**#	0.98±0.08	0.89±0.04**##

*P<0.05

**P<0.01 *vs* CON group

^#^*P*<0.05

^##^*P*<0.01 *vs* GDMN group.

#### Lower protein levels of Cav-1 in GDM-induced macrosomia

Placenta of GDM-induced macrosomia had significantly lower protein levels of Cav-1 compared to both CON and GDMN groups (p<0.05; Fig 2B). In addition, Cav-1 in GDMN group was lower than CON group (p<0.01; Fig 2B).

#### Increased proteins levels of GLUT1 and ACC and decreased proteins levels of p-AMPKα(Thr172) and p-ACC(Ser79) in GDM-induced macrosomia

Placenta in GDMM group owned the highest GLUT1 (Fig 2C).and ACC protein expression (Fig 2G). While, proteins levels of p-AMPKα(Thr172), p-ACC(Ser79) in GDMM group were all significantly lower than both GDMN and CON group (p<0.05; Fig 2D and 2E). Interestingly, compared with the CON group, the relative expression of GLUT1 protein in GDMN group was significantly increased (p<0.05; Fig 2C), while p-AMPKα(Thr172) and p-ACC(Ser79) protein expression was significantly decreased (p<0.05; Fig 2D and 2E). In addition, the total AMPKα protein levels was not significantly changed (Fig 2F).

### Relationship between the protein levels of Cav-1 and AMPK signaling pathway related placental proteins and NBW

**Fig 3:**
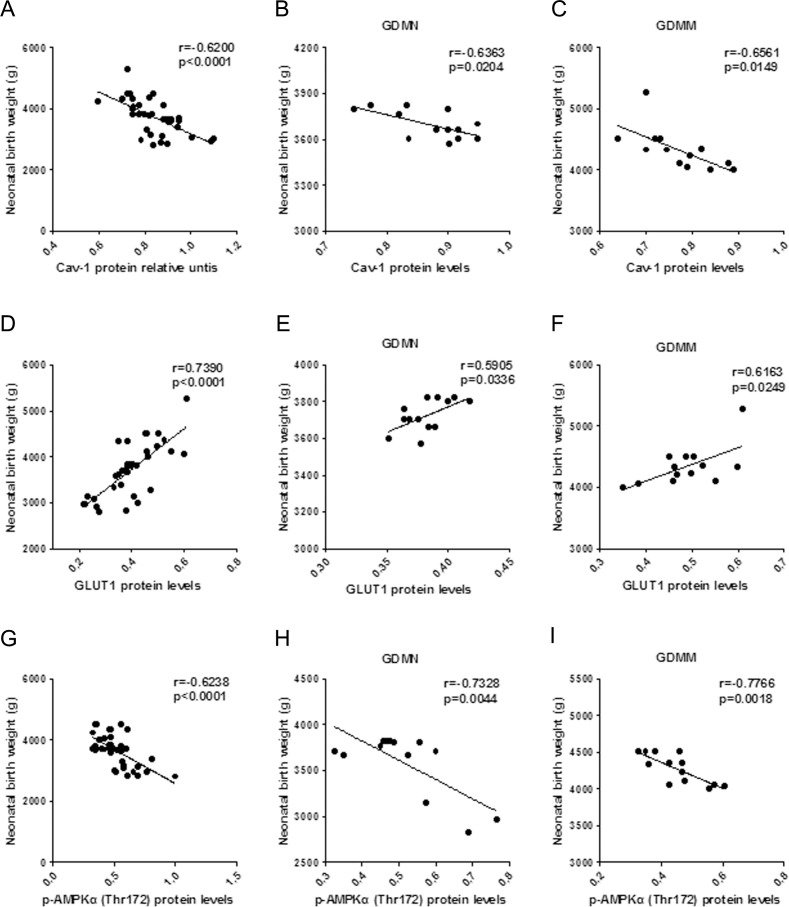
Relationship between NBW and placental protein levels of Cav-1, GLUT1 and p-AMPKα(Thr172). (A-C) Relationship between NBW and placental Cav-1 protein levels in all groups (A, n = 39), GDMN group (B, n = 13) and GDMM group (C, n = 13). (D-F) Relationship between NBW and GLUT1 protein levels in all groups (D, n = 39), GDMN group (E, n = 13) and GDMM group (F, n = 13). (G-I) Relationship between NBW and p-AMPKα(Thr172) protein levels in all placenta (G, n = 39), GDMN group (H, n = 13) and GDMM group (I, n = 13); r = Pearson’s correlation coefficient.

**Fig 4:**
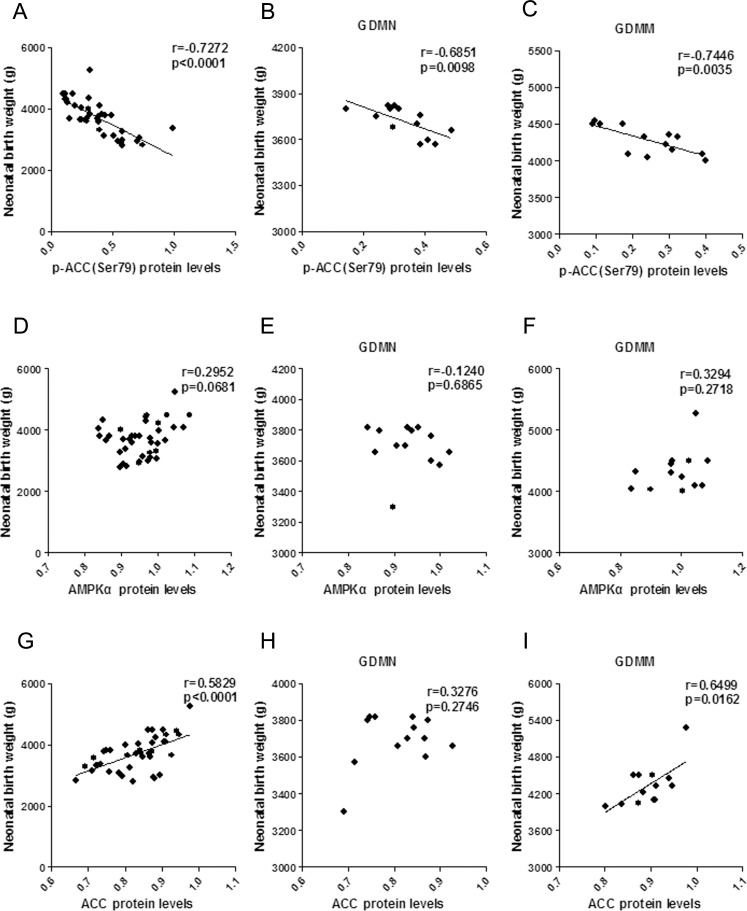
The relationship of between NBW and protein levels of p-ACC(Ser79), AMPKα and ACC. (A-C) Relationship between NBW and placental p-ACC(Ser79) protein levels in all groups (A, n = 39), GDMN group (B, n = 13) and GDMM group (C, n = 13). (D-F) Relationship between NBW and AMPKα protein levels in all groups (D, n = 39), GDMN group (E, n = 13) and GDMM group (F, n = 13). (G-I) Relationship between NBW and ACC protein levels in all placenta (G, n = 39), GDMN group (H, n = 13) and GDMM group (I, n = 13); r = Pearson’s correlation coefficient.

To evaluate the effects of alterations of Cav-1 and placental proteins associated with glucose and fatty acid metabolism on fetal growth, we performed Pearson correlation analysis on relationship of NBW and proteins levels. Our study showed that NBW was positively related to GLUT1 protein levels (p<0.05; Fig 3D–3F). While, Cav-1 (p<0.05; Fig 3A–3C), p-AMPKα(Thr172) (p<0.05; Fig 3G–3I) and p-ACC(Ser79) (p<0.05; Fig 4A–4C) were all have a negative relationship with NBW. Interestingly, there were no relationship between AMPKα with NBW, ACC protein levels had positive relationship with NBW in GDMM group, but not in GDMN group.

### Relationship between the protein levels of Cav-1 and AMPK signaling pathway related proteins

In order to further explore the role of Cav-1 in GDM-induced macrosomia, we analyzed the association of Cav-1 with the protein levels of GLUT1, p-AMPKα(Thr172) and p-ACC(Ser79).

#### Positive correlation between Cav-1 and p-AMPK, p-ACC

**Fig 5:**
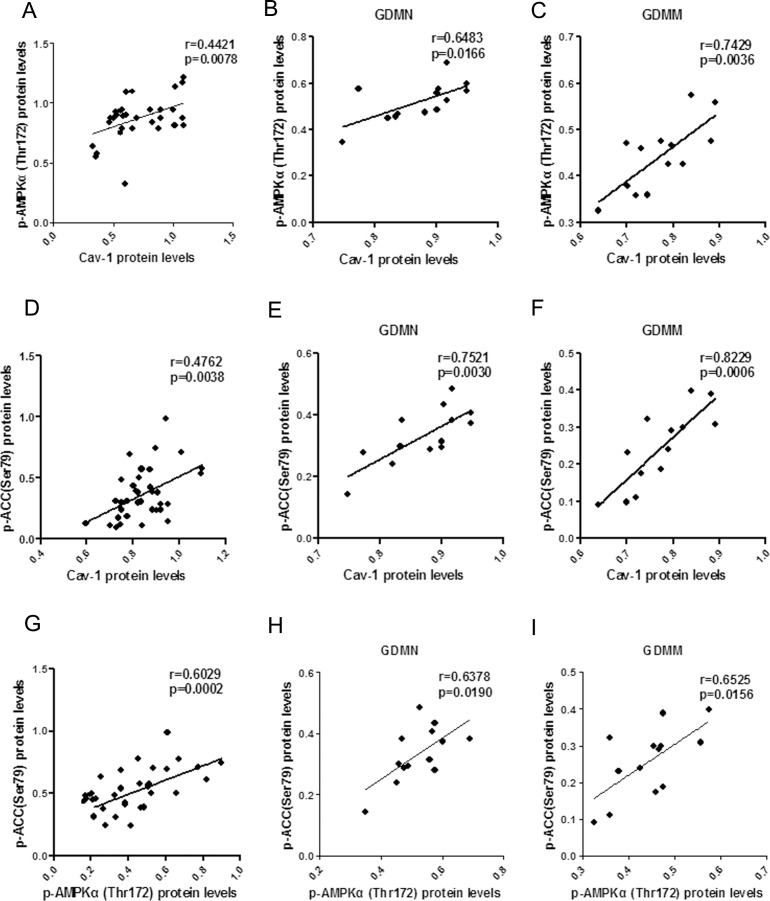
The relationship of p-AMPK, p-ACC and Cav-1 protein levels in placenta. (A-C) Respectively, Pearson correlation of Cav-1 with p-AMPK in all groups (A), GDMN group (B) and GDMM group (C). (D-F) Pearson correlation analysis of the relationship between Cav-1 and p-ACC in all groups (D), GDMN group (E) and GDMM group (F). (G-I) Relationship between p-AMPK and p-ACC protein levels in all placenta (G), GDMN group (H) and GDMM group (I).

In placenta, Both p-AMPK (Fig 5A–5C) and p-ACC (Fig 5D–5F) protein expression were positively associated with Cav-1 protein expression (p<0.05). A similar relation were also found between p-AMPK and p-ACC (p<0.05, Fig G-I).

#### Reversed correlation between Cav-1 and GLUT1

**Fig 6:**
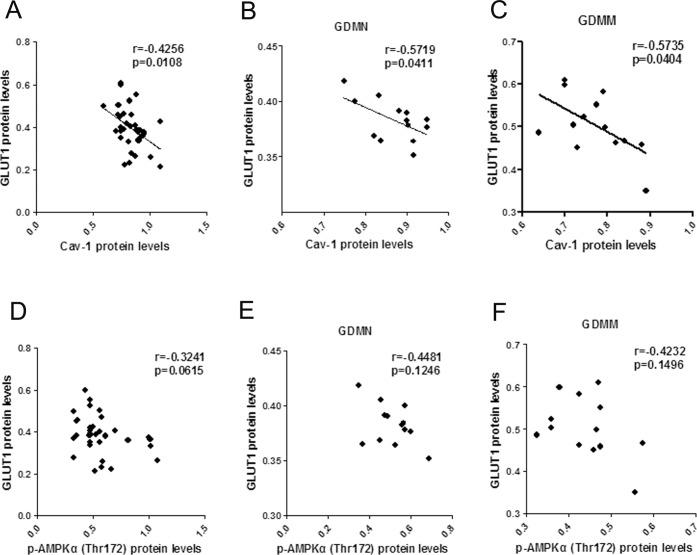
The relationship between Cav-1, p-AMPK and GLUT1 protein in placenta. (A-C) Pearson correlation analysis of Cav-1 protein levels versus GLUT1 in all group (A), GDMN group (B) and GDMM group (C). (D-F) p-AMPKα(Thr172) protein levels versus GLUT1 in all group (D), GDMN group (E) and GDMM group (F).

GLUT1 protein levels in placenta had a negative correlation with Cav-1 (p<0.05, Fig 6A–6C), but no significant correlation with p-AMPK (p>0.05, Fig 6D–6F).

### Alteration of mRNA levels of Cav-1 and AMPK signaling pathway related proteins in placenta

**Fig 7:**
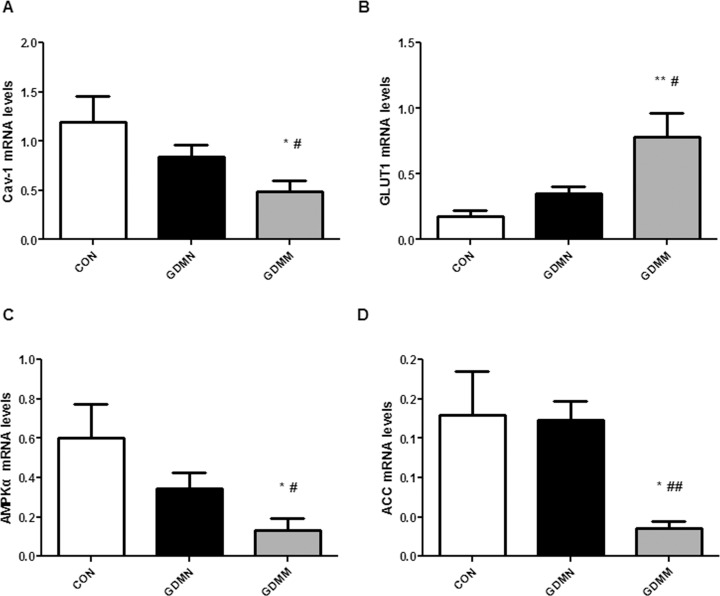
mRNA levels of Cav-1, GLUT1, ACC and AMPKα in placenta. Realtime-PCR analysis of relative mRNA levels of the genes from the four identified proteins in placenta. Bar graph shows mean ± SD of thirteen samples from each group. Cav-1 (A), GLUT1 (B), AMPKα (C) and ACC (D) indicated the gene names of Caveolin-1, Glucose transporter 1, Adenosine monophosphate-activated protein kinase α and Acetyl CoA carboxylase, respectively. Data displayed as Mean ± SD. *p<0.05, **p<0.01 vs CON group; #p<0.05, ##p<0.01vs GDMN group.

To compare the mRNA levels of GLUT1, Cav-1, ACC and AMPKα in GDM, GDMN and CON placenta, thirteen amples from each group were analyzed by realtime-PCR. As shown in Table 4 and Fig 7, the relative mRNA levels of Cav-1 were lowest in GDMM group (p<0.05; Fig 7A), with highest expression of GLUT1 (p<0.05; Fig 7B), inhibited ACC and AMPKα (p<0.05; Fig 7C and 7D) compared to both CON and GDMN groups. While there was no significant difference between the GDMN group and the CON group (p>0.05).

**Table 4:** mRNA levels of Cav-1, GLUT1, ACC and AMPKα in placenta.

	Cav-1 (n = 13)	GLUT1 (n = 13)	AMPKα (n = 13)	ACC (n = 13)
CON	1.23±0.25	0.17±0.05	0.60±0.17	0.18±0.05
GDMN	0.90±0.12	0.43±0.08	0.35±0.08	0.17±0.02
GDMM	0.50±0.10*^#^	0.78±0.18**^#^	0.13±0.06*^#^	0.04±0.01*^##^

*p<0.05

**p<0.01 *vs* CON group

^#^*p*<0.05

^##^*p*<0.01 *vs* GDMN group.

### Correlation analysis of NBW with mRNA levels of Cav-1, GLUT1, AMPKα and ACC

**Fig 8:**
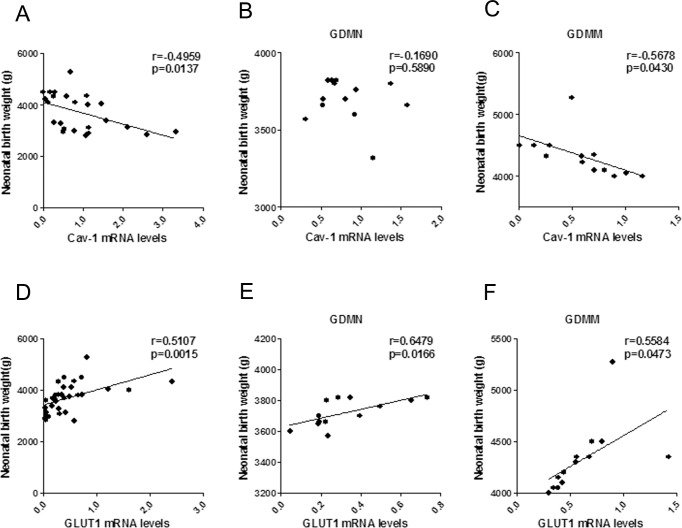
The association of Cav-1 and GLUT1 mRNA levels with NBW. (A-C) Respectively, Association of Cav-1 mRNA levels with NBW by Pearson correlation analysis in all groups (A; n = 39), GDMN group (B) and GDMM group (C). Positive relationship of GLUT1 mRNA levels and NBW by Pearson correlation analysis in all placenta (D), in GDMN group (E) and GDMM group (F).

**Fig 9:**
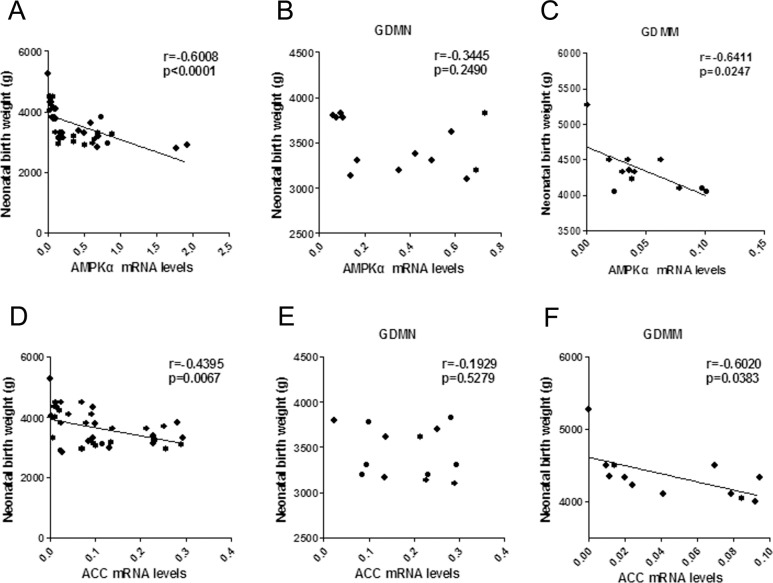
The association of AMPKα and ACC mRNA levels with NBW. No significant relationship between AMPKα mRNA levels with NBW in all groups (A, n = 39) or GDMN group (B) or GDMM group (C). Positive relationship of ACC mRNA levels and NBW by Pearson correlation analysis in all groups (D) and GDMM group (F), but not in GDMN group (E).

Correlation results in Fig 8 showed that NBW and placental Cav-1 mRNA levels was negatively correlated in GDMM group (r = -0.5678; p = 0.0430; Fig 8C), but not in GDMN group (p>0.05, Fig 8B). The correlation concerning mRNA of AMPK signaling pathway related proteins and NBW confirmed that GLUT1 mRNA levels was positively related to NBW (p<0.05, Fig 8D–8F). while, the mRNA levels of AMPKα (p<0.05; Fig 9A–9C) and ACC (p<0.05, Fig 9D–9F) were negatively related to NBW in GDMM group but not in GDMN group (p>0.05).

## Discussion

GDM has generally been acknowledged as a serious pregnancy complication to harm the fetus. Recent studies reveal macrosomia has a obviously rising trend among GDM-induced adverse pregnancy outcomes [20, 21]. Interestingly, no effective reduction of prevalence of macrosomia is achieved, although the clinical treatment of GDM has been significantly improved [22–24]. Previous studies mainly focused on improving maternal glucose homeostasis due to lower concentration difference of maternal-fetal glucose [25–27]. Our results indicated that the birth weight of GDM group was still significantly higher than that of the NGT group, in spite of achieving the ideal goal of maternal glycemic control in the third trimester. Combined with the correlation analysis results, there is no doubt that GDM patients have a more important risk factor in the induction of macrosomia than maternal glucose levels.

In addition to concentration difference of maternal-fetal placenta, the levels of the relevant placental transport proteins is also a dominant factor in placental glucose and fatty acid transports [28], and closely related to the development of fetal macrosomia [29, 30]. Given the role of Cav-1 and AMPK signaling pathway related proteins in glucose and lipid metabolism, it is important to identify and analyze both the mRNA and protein expression of Cav-1, AMPKα, GLUT1 and ACC in placenta of GDMM, GDMN and CON group and their relationship with NBW.

Strong evidence indicates that GLUT1 is the rate-limiting step in placental glucose transport, thus alteration in GLUT1 expression will have a marked impact on the glucose transport and fetal growth [31, 32]. Collectively, GLUT1 is the key point of the study on GDM placental glucose transport. Just as we conceived, the dysbolism of GDM indeed induced the activation of GLUT1 protein and mRNA expression in placenta. Given the positive correlation between GLUT1 and NBW, we conclude that GLUT1 makes a potential contribution of placental glucose transport to newborn weight in GDM, which may be part of the reason why GDM-induced macrosomias still significantly increase through improving maternal glycemic control in the late pregnancy.

ACC, as the rate limiting enzyme, plays a vital role in the synthesis of fatty acid, mainly phosphorylated by the upstream factor p-AMPK to inhibit fatty acid activation and promote fatty acid oxidation to achieve the reduction of the fatty acid synthesis [33–36]. By evaluating the alterations of placenta glucose and fatty acid metabolism related factors, we found that the mRNA and phosphorylation of ACC in GDMM group were significantly inhibited. At the same time, ACC protein levels was increased. Given the association with NBW, we can conjecture that the decreased p-ACC and increased ACC result in the induction of macrosomia via excessive fatty acid synthesis to enhance placental fatty acid transport.

Cav-1 is widely involved in glucose metabolism and closely related to the regulation of the expression of glucose transporters [37–39]. The data we collected confirmed that GDM-induced macrosomia had the lowest mRNA and protein levels of Cav-1 and p-AMPK. Moreover, we reported a negative association of Cav-1 vensus GLUT1 and a positive correlation of Cav-1 with p-AMPK, consistent with the role of Cav-1 on AMPK in previous studies. But there was no significant correlation between p-AMPK and GLUT1 protein, which indicated that Cav-1 participates in the process of the reduction of GLUT1 expression independently of the AMPK signaling pathway. Combining with negative relevant relations to NBW, it is reasonable to make a conclusion that Cav-1 may be involved in GDM-induced macrosomia via reduction of GLUT1-mediated glucose metabolism.

In view of the close relationship between Cav-1 and lipid metabolism [40], we explored the underlying relationship between Cav-1 and AMPK-mediated fatty acid metabolism in placenta. Our findings showed that Cav-1, p-AMPK and p-ACC were all significantly decreased in GDMM placenta. And Cav-1 was positively associated with p-ACC and p-AMPK protein, which indicated that phosphorylation levels of AMPK and ACC were all decreased with the decreased Cav-1 in placenta, result in excessive placental fatty acid transport. Meanwhile, positive relationship of p-AMPK and p-ACC revealed that the activation of ACC was depended on AMPK signaling pathway. Combined with the positive correlation between Cav-1 and p-AMPK, we conclude that Cav-1 can induce AMPK-mediated lipid metabolism to participate in GDM-induced macrosomia.

In conclusion, our study showed that the protein and mRNA levels of Cav-1 were significantly inhibited in the placenta of GDM-induced macrosomia. Moreover, Cav-1 may involve in GDM-induced macrosomia via the induction of AMPK-mediated placental fatty acid metabolism and the reduction of GLUT1-mediated placental glucose metabolism. However, further research is needed to study the exact mechanism of the Cav-1 in the pathogenesis of GDM and larger studies will be required to clarify the role of Cav-1 on AMPK signaling pathway.

## Supporting Information

S1 Dataset p-AMPKα(Thr172) protein levels in three groups. (PZF)

S2 Dataset GLUT1 protein levels in three groups. (PZF)

S3 Dataset Cav-1 protein levels in three groups. (PZF)

S1 Fig Ethics Statement. (TIF)

S2 Fig Ethics Statement. (TIF)

S3 Fig Ethics Statement. (TIF)

S4 Fig informed consent. (TIF)

S5 Fig informed consent. (TIF)

## References

[1] Karantanos T, Karanika S, Wang J, Yang G, Dobashi M, Park S, et al Caveolin-1 regulates hormone resistance through lipid synthesis, creating novel therapeutic opportunities for castration-resistant prostate cancer. Oncotarget. 2016. Epub 2016/06/23. 10.18632/oncotarget.10113 PMC5216801 27331874

[2] Li J, Song L, Zhou L, Wu J, Sheng C, Chen H, et al A MicroRNA Signature in Gestational Diabetes Mellitus Associated with Risk of Macrosomia. Cellular physiology and biochemistry: international journal of experimental cellular physiology, biochemistry, and pharmacology. 2015;37(1):243–52. Epub 2015/08/26. 10.1159/000430349 26302821

[3] Wahlberg J, Ekman B, Nystrom L, Hanson U, Persson B, Arnqvist HJ. Gestational diabetes: Glycaemic predictors for fetal macrosomia and maternal risk of future diabetes. Diabetes research and clinical practice. 2016;114:99–105. Epub 2016/01/29. 10.1016/j.diabres.2015.12.017 26818892

[4] Zhu M, Cai J, Liu S, Huang M, Chen Y, Lai X, et al Relationship between gestational fasting plasma glucose and neonatal birth weight, prenatal blood pressure and dystocia in pregnant Chinese women. Diabetes/metabolism research and reviews. 2014;30(6):489–96. Epub 2014/03/26. 10.1002/dmrr.2544 24665054

[5] Huo Y, Liu SX, Song GY, Ren LP, Wang C, Zhang DH. Plasma levels and placental expression of vaspin in pregnant women with diabetes mellitus. Brazilian journal of medical and biological research = Revista brasileira de pesquisas medicas e biologicas / Sociedade Brasileira de Biofisica [et al]. 2015;48(3):273–9. Epub 2015/01/22. 10.1590/1414-431X20143432 PMC4381949 25608237

[6] Mai C, Hou M, Chen R, Duan D, Xu H, Lin X, et al Cardiovascular risk factors in Chinese women with a history of gestational diabetes mellitus. International journal of clinical and experimental medicine. 2015;8(11):21694–8. Epub 2016/02/18. 26885128 PMC4723973

[7] Wilson BL, Dyer JM, Latendresse G, Wong B, Baksh L. Exploring the Psychosocial Predictors of Gestational Diabetes and Birth Weight. Journal of obstetric, gynecologic, and neonatal nursing: JOGNN / NAACOG. 2015;44(6):760–71. Epub 2015/09/25. 10.1111/1552-6909.12754 26402777

[8] King RG, Osmond DT, Brennecke SP, Gude NM. Effect of fetal macrosomia on human placental glucose transport and utilization in insulin-treated gestational diabetes. Journal of perinatal medicine. 2003;31(6):475–83. Epub 2004/01/09. 10.1515/JPM.2003.073 14711103

[9] Park S, Kang S, Jeong DY, Jeong SY, Park JJ, Yun HS. Cyanidin and malvidin in aqueous extracts of black carrots fermented with Aspergillus oryzae prevent the impairment of energy, lipid and glucose metabolism in estrogen-deficient rats by AMPK activation. Genes & nutrition. 2015;10(2):455. Epub 2015/02/24. 25701199 10.1007/s12263-015-0455-5 PMC4336299

[10] Gaccioli F, White V, Capobianco E, Powell TL, Jawerbaum A, Jansson T. Maternal overweight induced by a diet with high content of saturated fat activates placental mTOR and eIF2alpha signaling and increases fetal growth in rats. Biology of reproduction. 2013;89(4):96 Epub 2013/09/06. 10.1095/biolreprod.113.109702 24006279

[11] Martino J, Sebert S, Segura MT, Garcia-Valdes L, Florido J, Padilla MC, et al Maternal Body Weight and Gestational Diabetes Differentially Influence Placental and Pregnancy Outcomes. The Journal of clinical endocrinology and metabolism. 2016;101(1):59–68. Epub 2015/10/30. 10.1210/jc.2015-2590 26513002 PMC4701853

[12] Muir R, Ballan J, Clifford B, McMullen S, Khan R, Shmygol A, et al Modelling maternal obesity: the effects of a chronic high-fat, high-cholesterol diet on uterine expression of contractile-associated proteins and ex vivo contractile activity during labour in the rat. Clin Sci (Lond). 2016;130(3):183–92. Epub 2015/11/07. 26543049 10.1042/CS20150539 PMC4682211

[13] Peng XL, Qu W, Wang LZ, Huang BQ, Ying CJ, Sun XF, et al Resveratrol ameliorates high glucose and high-fat/sucrose diet-induced vascular hyperpermeability involving Cav-1/eNOS regulation. PloS one. 2014;9(11):e113716 Epub 2014/11/25. 10.1371/journal.pone.0113716 25419974 PMC4242725

[14] Zhang QQ, Huang L, Han C, Guan X, Wang YJ, Liu J, et al Caveolin-1 and glucose transporter 4 involved in the regulation of glucose-deprivation stress in PC12 cells. Sheng li xue bao: [Acta physiologica Sinica]. 2015;67(4):349–56. Epub 2015/08/25. 26300246

[15] Zhang W, Wang K, Yu W, Liu Y, Chu J, Jiang Y, et al [Diagnostic utility of S100A1, GLUT-1 and Caveolin-1 in renal tumors with oncocytic features: a comparative study]. Zhonghua bing li xue za zhi = Chinese journal of pathology. 2015;44(11):767–71. Epub 2016/02/19. 26888385

[16] Fernandez-Real JM, Catalan V, Moreno-Navarrete JM, Gomez-Ambrosi J, Ortega FJ, Rodriguez-Hermosa JI, et al Study of caveolin-1 gene expression in whole adipose tissue and its subfractions and during differentiation of human adipocytes. Nutrition & metabolism. 2010;7:20. Epub 2010/03/17. 20226013 10.1186/1743-7075-7-20 PMC2858724

[17] Oh YS, Kim HJ, Ryu SJ, Cho KA, Park YS, Park H, et al Exercise type and muscle fiber specific induction of caveolin-1 expression for insulin sensitivity of skeletal muscle. Experimental & molecular medicine. 2007;39(3):395–401. Epub 2007/07/03. 17603294 10.1038/emm.2007.44

[18] Penumathsa SV, Thirunavukkarasu M, Zhan L, Maulik G, Menon VP, Bagchi D, et al Resveratrol enhances GLUT-4 translocation to the caveolar lipid raft fractions through AMPK/Akt/eNOS signalling pathway in diabetic myocardium. Journal of cellular and molecular medicine. 2008;12(6A):2350–61. Epub 2008/02/13. 10.1111/j.1582-4934.2008.00251.x 18266981 PMC4514113

[19] Sun D, Li F, Zhang Y, Xu X. Associations of the pre-pregnancy BMI and gestational BMI gain with pregnancy outcomes in Chinese women with gestational diabetes mellitus. International journal of clinical and experimental medicine. 2014;7(12):5784–9. Epub 2015/02/11. 25664107 PMC4307554

[20] Mirzamoradi M, Heidar Z, Faalpoor Z, Naeiji Z, Jamali R. Comparison of glyburide and insulin in women with gestational diabetes mellitus and associated perinatal outcome: a randomized clinical trial. Acta medica Iranica. 2015;53(2):97–103. Epub 2015/03/01. 25725178

[21] Nobles C, Marcus BH, Stanek EJ 3rd, Braun B, Whitcomb BW, Solomon CG, et al Effect of an exercise intervention on gestational diabetes mellitus: a randomized controlled trial. Obstetrics and gynecology. 2015;125(5):1195–204. Epub 2015/05/02. 10.1097/AOG.0000000000000738 25932848 PMC4418021

[22] Brett KE, Ferraro ZM, Holcik M, Adamo KB. Placenta nutrient transport-related gene expression: the impact of maternal obesity and excessive gestational weight gain. The journal of maternal-fetal & neonatal medicine: the official journal of the European Association of Perinatal Medicine, the Federation of Asia and Oceania Perinatal Societies, the International Society of Perinatal Obstet. 2016;29(9):1399–405. Epub 2015/06/13. 10.3109/14767058.2015.1049522 26067267

[23] Jarmuzek P, Wielgos M, Bomba-Opon D. Placental pathologic changes in gestational diabetes mellitus. Neuro endocrinology letters. 2015;36(2):101–5. Epub 2015/06/14. 26071574

[24] Su R, Wang C, Feng H, Lin L, Liu X, Wei Y, et al Alteration in Expression and Methylation of IGF2/H19 in Placenta and Umbilical Cord Blood Are Associated with Macrosomia Exposed to Intrauterine Hyperglycemia. PloS one. 2016;11(2):e0148399 Epub 2016/02/04. 10.1371/journal.pone.0148399 26840070 PMC4739655

[25] Briley AL, Barr S, Badger S, Bell R, Croker H, Godfrey KM, et al A complex intervention to improve pregnancy outcome in obese women; the UPBEAT randomised controlled trial. BMC pregnancy and childbirth. 2014;14:74 Epub 2014/02/19. 10.1186/1471-2393-14-74 24533897 PMC3938821

[26] Hernandez TL, Van Pelt RE, Anderson MA, Daniels LJ, West NA, Donahoo WT, et al A higher-complex carbohydrate diet in gestational diabetes mellitus achieves glucose targets and lowers postprandial lipids: a randomized crossover study. Diabetes care. 2014;37(5):1254–62. Epub 2014/03/07. 10.2337/dc13-2411 24595632 PMC3994935

[27] Javadian P, Alimohamadi S, Gharedaghi MH, Hantoushzadeh S. Gestational diabetes mellitus and iron supplement; effects on pregnancy outcome. Acta medica Iranica. 2014;52(5):385–9. Epub 2014/06/06. 24902020

[28] Holme AM, Roland MC, Lorentzen B, Michelsen TM, Henriksen T. Placental glucose transfer: a human in vivo study. PloS one. 2015;10(2):e0117084 Epub 2015/02/14. 10.1371/journal.pone.0117084 25680194 PMC4334523

[29] Brett KE, Ferraro ZM, Yockell-Lelievre J, Gruslin A, Adamo KB. Maternal-fetal nutrient transport in pregnancy pathologies: the role of the placenta. International journal of molecular sciences. 2014;15(9):16153–85. Epub 2014/09/16. 10.3390/ijms150916153 25222554 PMC4200776

[30] Larque E, Pagan A, Prieto MT, Blanco JE, Gil-Sanchez A, Zornoza-Moreno M, et al Placental fatty acid transfer: a key factor in fetal growth. Annals of nutrition & metabolism. 2014;64(3–4):247–53. Epub 2014/10/11. 25300267 10.1159/000365028

[31] Baumann MU, Schneider H, Malek A, Palta V, Surbek DV, Sager R, et al Regulation of human trophoblast GLUT1 glucose transporter by insulin-like growth factor I (IGF-I). PloS one. 2014;9(8):e106037 Epub 2014/08/27. 10.1371/journal.pone.0106037 25157747 PMC4144961

[32] Sciullo E, Cardellini G, Baroni MG, Torresi P, Buongiorno A, Pozzilli P, et al Glucose transporter (Glut1, Glut3) mRNA in human placenta of diabetic and non-diabetic pregnancies. Early pregnancy: biology and medicine: the official journal of the Society for the Investigation of Early Pregnancy. 1997;3(3):172–82. Epub 1999/03/23. 10086067

[33] Lee H, Kang R, Bae S, Yoon Y. AICAR, an activator of AMPK, inhibits adipogenesis via the WNT/beta-catenin pathway in 3T3-L1 adipocytes. International journal of molecular medicine. 2011;28(1):65–71. Epub 2011/04/15. 10.3892/ijmm.2011.674 21491080

[34] Lennon JC, Butini S, Campiani G, O'Meara A, Williams DC, Zisterer DM. Involvement of AMP-activated protein kinase in mediating pyrrolo-1,5-benzoxazepine-induced apoptosis in neuroblastoma cells. Investigational new drugs. 2016;34(5):663–76. Epub 2016/06/24. 10.1007/s10637-016-0366-3 27334143

[35] Madiraju AK, Alves T, Zhao X, Cline GW, Zhang D, Bhanot S, et al Argininosuccinate synthetase regulates hepatic AMPK linking protein catabolism and ureagenesis to hepatic lipid metabolism. Proceedings of the National Academy of Sciences of the United States of America. 2016;113(24):E3423–30. Epub 2016/06/02. 10.1073/pnas.1606022113 27247419 PMC4914193

[36] Wysham WZ, Roque DR, Han J, Zhang L, Guo H, Gehrig PA, et al Effects of Fatty Acid Synthase Inhibition by Orlistat on Proliferation of Endometrial Cancer Cell Lines. Targeted oncology. 2016. Epub 2016/05/18. 10.1007/s11523-016-0442-9 27188391

[37] Codenotti S, Vezzoli M, Poliani PL, Cominelli M, Bono F, Kabbout H, et al Caveolin-1, Caveolin-2 and Cavin-1 are strong predictors of adipogenic differentiation in human tumors and cell lines of liposarcoma. European journal of cell biology. 2016;95(8):252–64. Epub 2016/05/12. 10.1016/j.ejcb.2016.04.005 27168348

[38] Kim HS, Kim HJ, Kim YS, Park SC, Harris R, Kim CK. Caveolin, GLUT4 and insulin receptor protein content in human arm and leg muscles. European journal of applied physiology. 2009;106(2):173–9. Epub 2009/02/17. 10.1007/s00421-009-1001-1 19219452

[39] Palacios-Ortega S, Varela-Guruceaga M, Milagro FI, Martinez JA, de Miguel C. Expression of Caveolin 1 is enhanced by DNA demethylation during adipocyte differentiation. status of insulin signaling. PloS one. 2014;9(4):e95100 Epub 2014/04/23. 10.1371/journal.pone.0095100 24751908 PMC3994010

[40] Salis O, Bedir A, Ozdemir T, Okuyucu A, Alacam H. The relationship between anticancer effect of metformin and the transcriptional regulation of certain genes (CHOP, CAV-1, HO-1, SGK-1 and Par-4) on MCF-7 cell line. European review for medical and pharmacological sciences. 2014;18(11):1602–9. Epub 2014/06/20. 24943970

