# Supplementary figures and images for: GDM-Induced Macrosomia Is Reversed by Cav-1 via AMPK-Mediated Fatty Acid Transport and GLUT1-Mediated Glucose Transport in Placenta
Source: PLoS One. 2017 Jan 26;12(1):e0170490. doi: 10.1371/journal.pone.0170490 (PMC5268469; doi:10.1371/journal.pone.0170490)

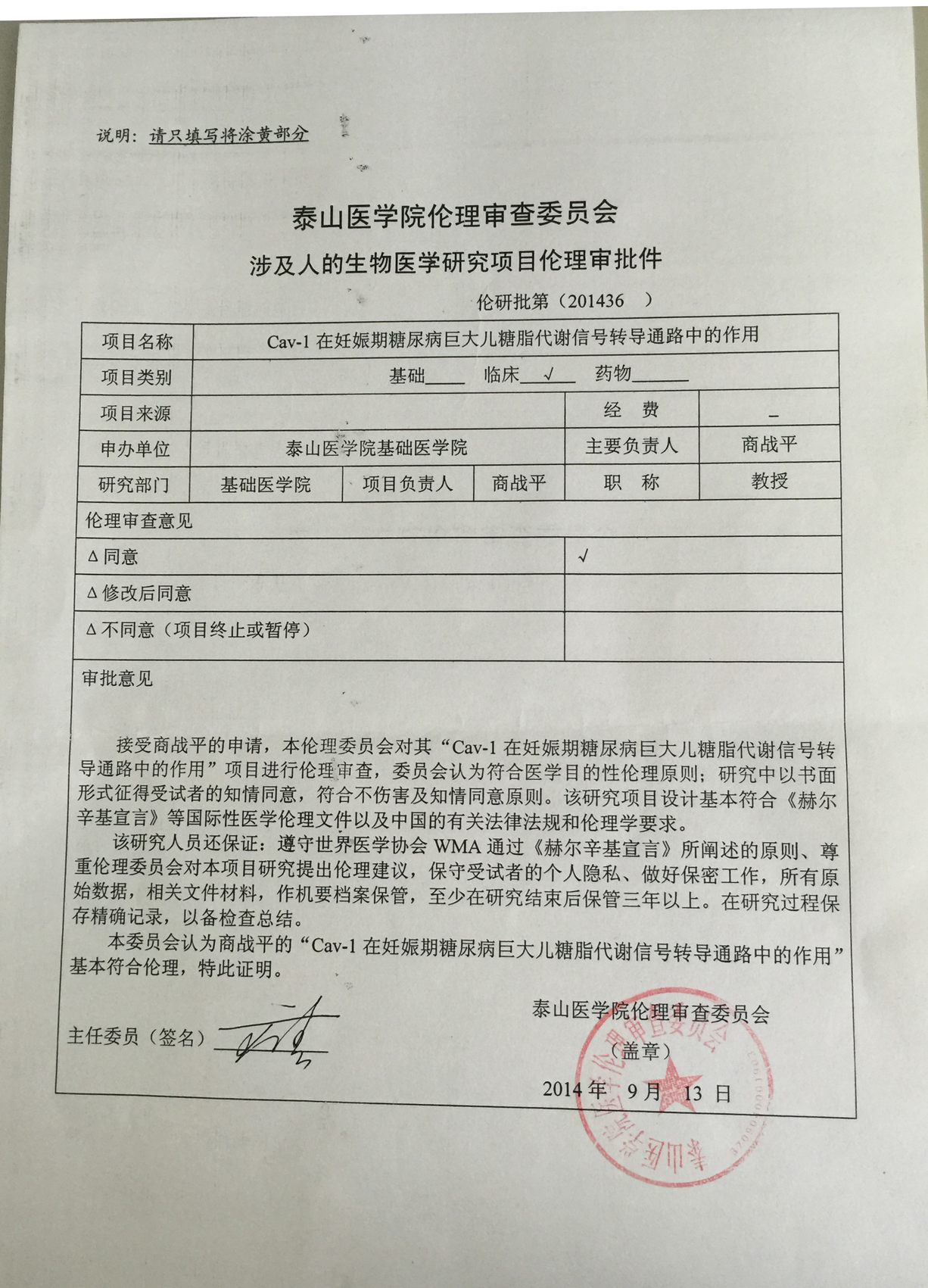

Supplement: S1 Fig — (TIF) [file pone.0170490.s004.tif]

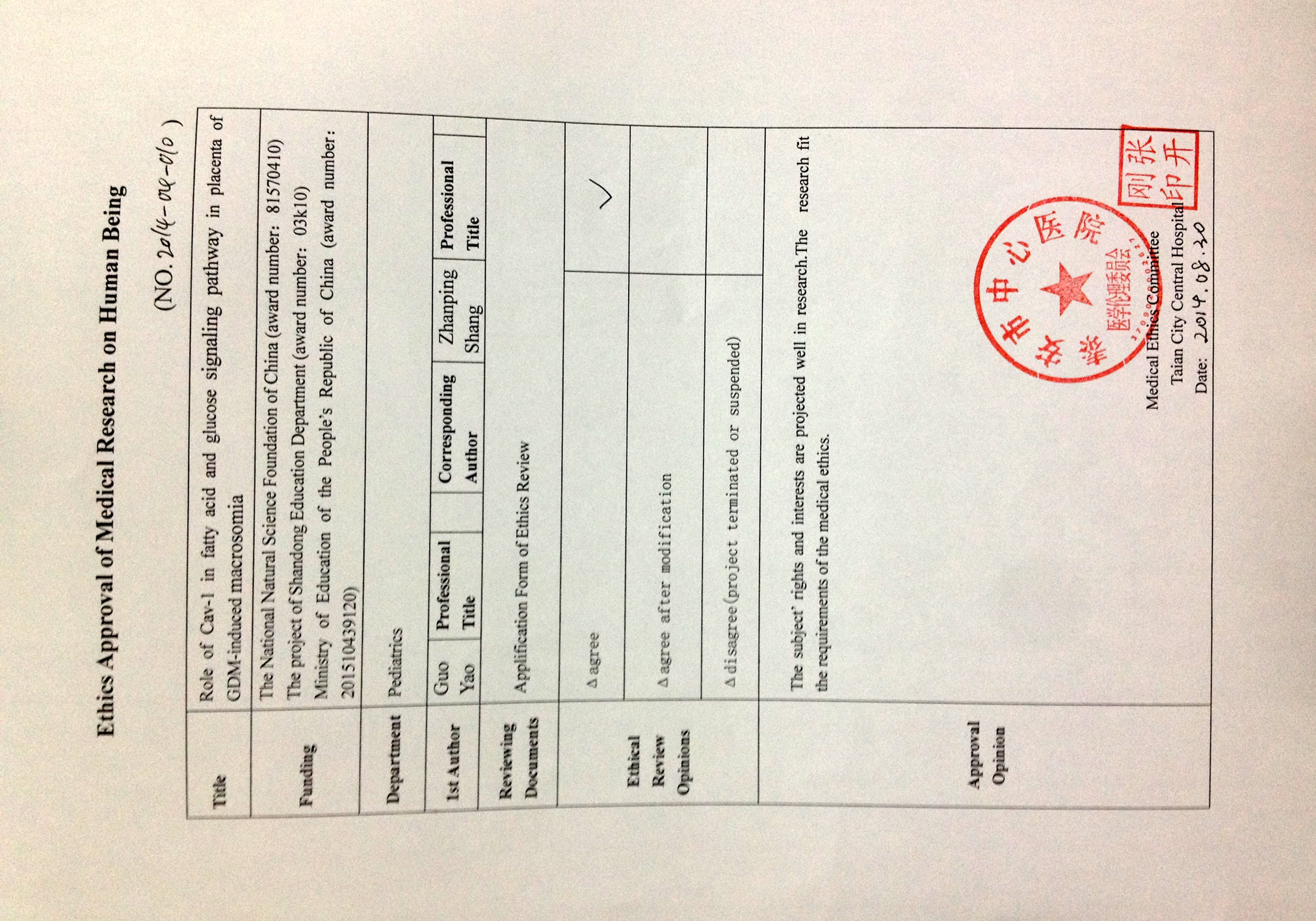

Supplement: S2 Fig — (TIF) [file pone.0170490.s005.tif]

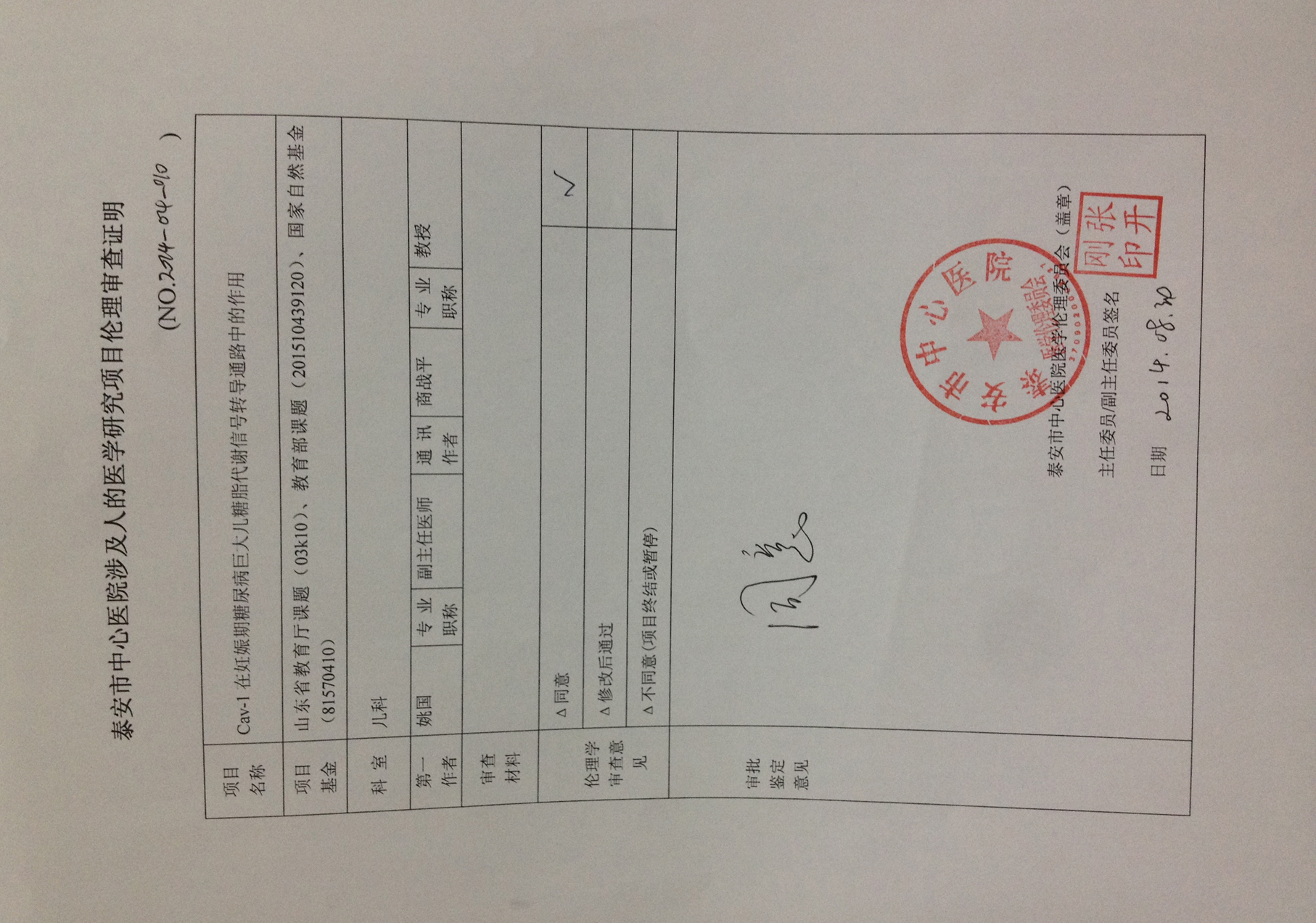

Supplement: S3 Fig — (TIF) [file pone.0170490.s006.tif]

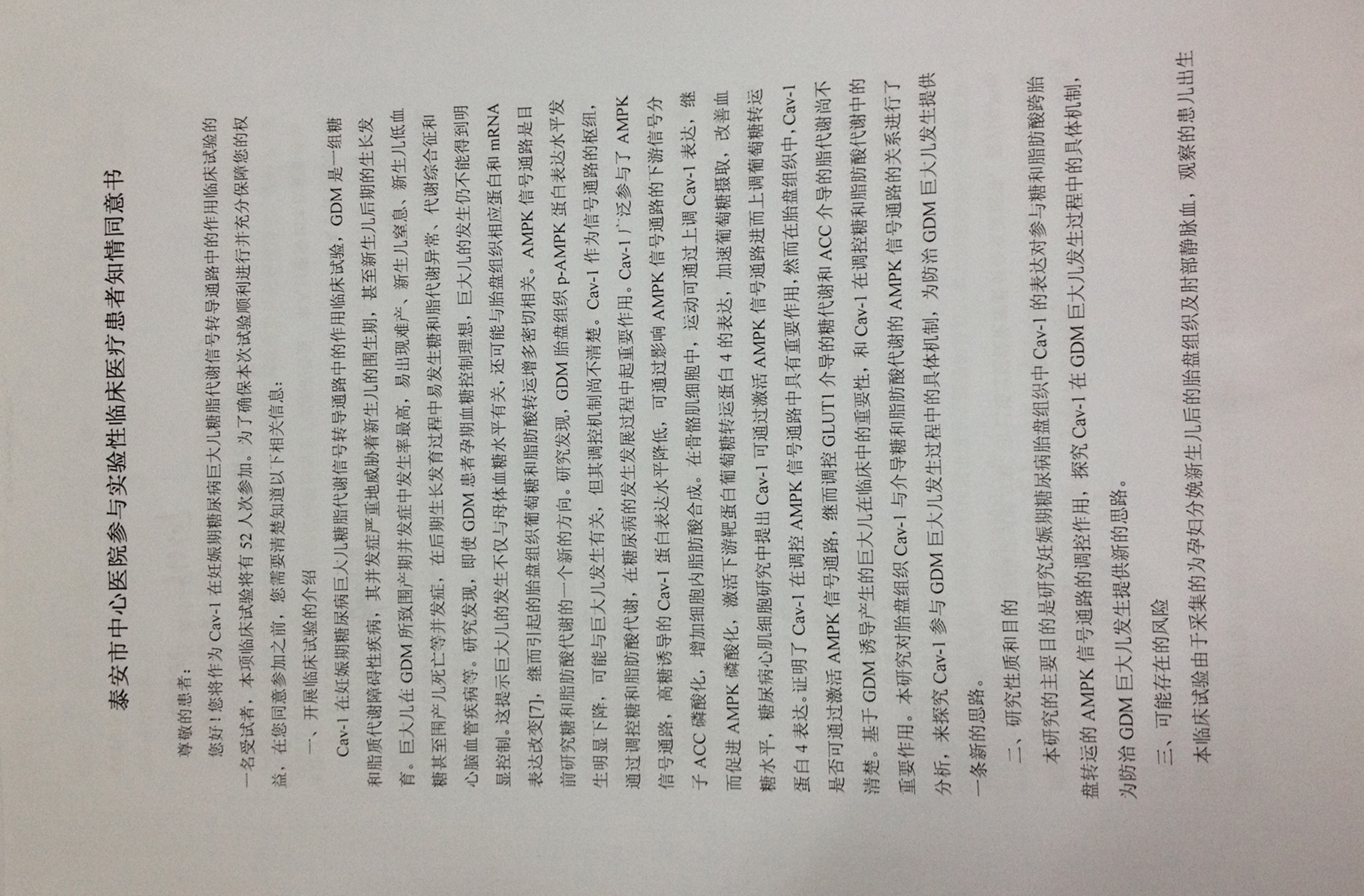

Supplement: S4 Fig — (TIF) [file pone.0170490.s007.tif]

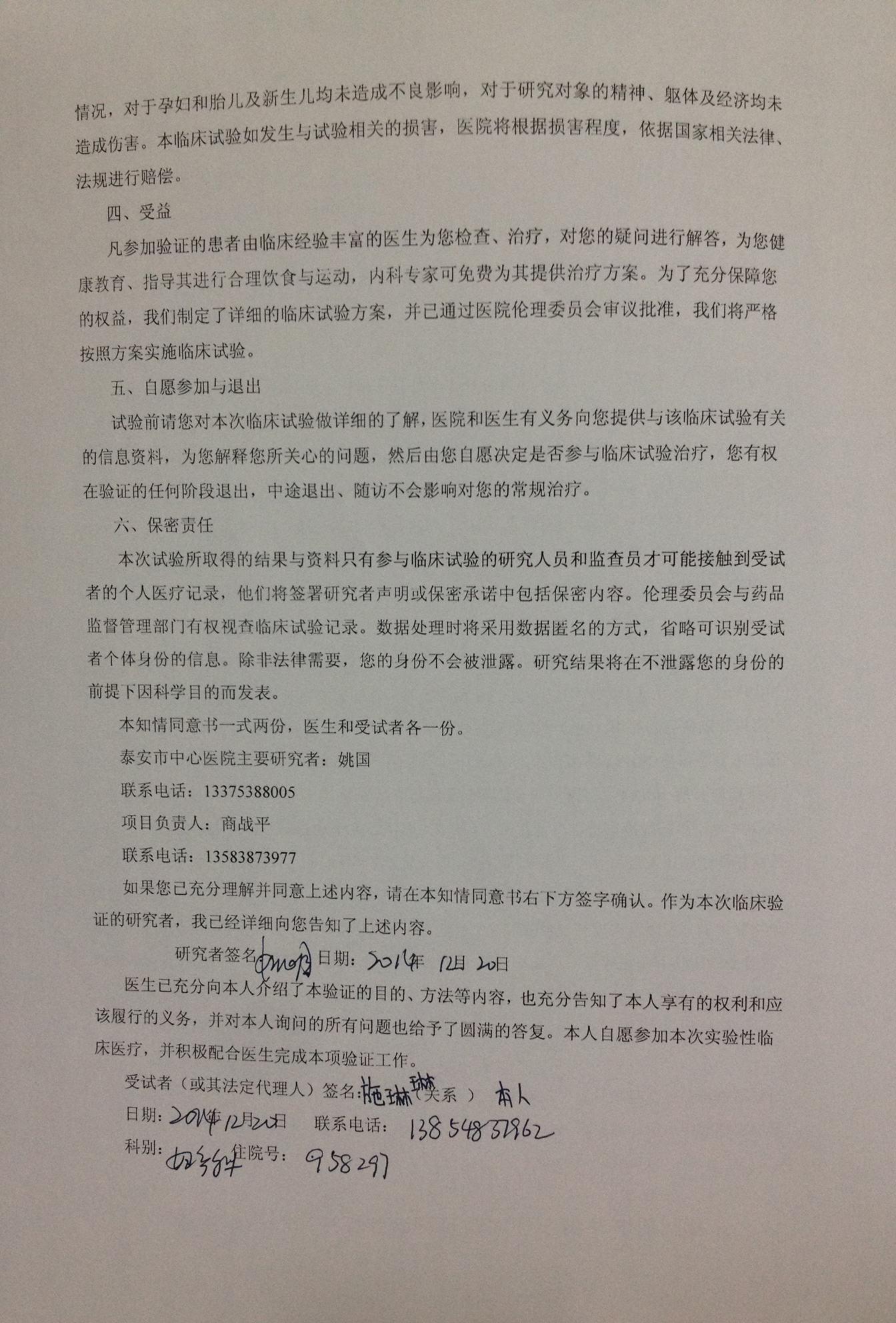

Supplement: S5 Fig — (TIF) [file pone.0170490.s008.tif]
